# Subrenal Capsule Assay - SRCA: The Promising Re-Emergence of a Long-Forgotten Method in Preclinical Nuclear Medical Cancer Diagnostics

**DOI:** 10.7150/jca.78599

**Published:** 2023-01-01

**Authors:** Zita Képes, György Trencsényi

**Affiliations:** Division of Nuclear Medicine and Translational Imaging, Department of Medical Imaging, Faculty of Medicine, University of Debrecen, Nagyerdei St. 98, H-4032 Debrecen, Hungary.

**Keywords:** Metastasis, Parathymic Lymph Node, Positron Emission Tomography, Subrenal Capsule Assay, Transplantation, Tumorigenesis

## Abstract

Since metastases are responsible for the majority of cancer-related death, in-depth understanding of metastatisation may provide an opportunity for the introduction of new therapeutic as well as diagnostic approaches in cancer management. Previously, preclinical research into the development and progression of malignancies has been hampered by the relative paucity of *in vivo* tumor models. Subrenal capsule assay (SRCA)-induced tumor-bearing experimental animal models, however, serve as potential preclinical model systems for the assessment of primary tumors and the route of metastatic spread. Several studies implementing either Hepatocarcinoma (He/De), Mesoblastic Nephroma (Ne/De), chemically induced Myeloid Leukemia (My1/De), or spontaneous Myeloid Myelomonocytic Leukemia (My2/De) cells under the left renal capsule of rats applying SRCA method were underway to study secondary tumor formation. Based on the results of this research, subrenally transplanted neoplastic cells led to the appearance of metastasis in the parathymic lymph nodes. Therefore, renal capsule/parathymic lymph node complex seems to be valuable in the *in vivo* evaluation of the process of metastatisation and the occurrence of secondary neoplasms. The re-emergence of this highly promising SRCA-based renal capsule/parathymic lymph node complex in preclinical nuclear medicine and experimental oncology may open a novel field towards drug trials and radiopharmaceutical development. In the present review, we provide a detailed overview of the history of SRCA as well as the series of research on the establishment of renal capsule/parathymic lymph node complex.

## Introduction

Preclinical studies have a crucial role in the development of novel molecules for either diagnostic or therapeutic purposes. To determine the suitability of a new compound - such as a diagnostic agent - for human application profound research studies and tests must take place at preclinical level prior to the clinical examinations. Within the framework of preclinical analysis *in vitro* cell systems and *in vivo* animal models are applied to assess the molecule in concern. Unfortunately, translational research is hampered by the relative paucity of *in vitro* molecular biological methods that are suitable for the evaluation of all phases of the development of different diseases for example the metastatic capability of various tumors. Further, *in vitro* tests are not appropriate to precisely model the effects of a new molecule or a drug-to-be on human organisms, their biodistribution, or their involvement in metabolic processes. Therefore, *in vivo* experiments and animal model systems are required to adequately investigate the pharmacokinetic characteristics of a drug candidate or a new radiolabelled diagnostic agent. Experimental animal models are of pivotal importance in basic oncological research, however, given the complexity of tumorigenesis and metastases formation, the selection of the best fitting model seems to be challenging. Out of the broadly available translational oncological experimental models, Subrenal Capsule Assay (SRCA)-induced rat metastasis system is supposed to be the least widespread. In this present review we aimed at outlining the significance of SRCA-based metastasis model that may be a promising tool in the synthesis of novel, tumor-specific radiopharmaceuticals in the field of nuclear medicine.

## The history and efficacy of the original SRCA method

### Early animal models

SRCA has been developed by *Bogden* in 1978 to test the efficacy of chemotherapeutic agents in human tumor xenografts 1, 2. Taking the maintenance of cell membrane integrity, cell-to-cell contact, and the spatial relationship of the cell populations and the tissues into account, SRCA seemed to be promising regarding both the therapeutic evaluation of new anticancer drugs and the assessment of prospective tumor sensitivity 2.

In their initial experiences a volume of 1 mm^3^ tumor sample (1×1×1 mm specimen) was transplanted under the left renal capsule of nude athymic mice 3. Tumor fragments comprising 70% of neoplastic cells are recommended for the assay 2. Given the permeability of this tiny fragment size, appropriate nourishment without the necessity for the development of a new tumor vasculature is granted 4. This abbreviates the lag time prior to the appearance of determinable tumor growth 4. The abundantly vascularised subrenal niche provides satisfactory nutrient and oxygen supply for the tumor, therefore, it ensures tumor progression and the delivery of the investigated drugs as well 3. Further, the transparency of the renal capsule makes the tumor easily visible 4. Sometimes it takes more time for the tumor to adapt to the microenvironment of the area under the renal capsule, consequently entire tumor formation could not be completed within 24 hours post-implantation 3. Given the characteristics of the implantation, histological data strengthened that the tumor forming procedure may take one-to-two days to be completed after implantation 5. As a result, analysis performed on day 1 or 2 should be handled cautiously since early results may indicate anti-implantation effects of the tested drugs instead of anti-maturation 5. In the research series of Bogden *et al.,* nude mice were administered treatment on day 1-10 and measurements of the implanted xenograft were performed on the day of the transplantation and on the 11^th^ day of the assay 3. Prior data strengthened the suitability of the 11-day long time-window employed in the original SRCA technique concerning the determination of tumor size and the extent of regression 4. The 1 mm^3^ specimen size, the subrenal area with its rich vascularity, and the adequate technique permitting *in situ* measurements were pivotal for gaining genuine results 4.

### Immunological aspects of the early models

Later, xenograft associated host immune response drew attention to the establishment of a shortened assay time frame. As primary antigenic stimulus appears between the 7^th^ and the 10^th^ day, and 9-to-12 days are required for the occurrence of cell-mediated responses, SRCA would have to be conducted within 6 days 4. However, the rate of development of the implanted tumor, and the capability of the measurement techniques to detect minor alterations largely determines the performance of the 6-day assay 4. Normal host immune reaction corresponds to the size of the transplanted xenograft and its existence till the termination of the test 2. In addition, host cell ingrowth could be triggered by injuries to the graft 2. The histological structure of the tumor implanted either in athymic or immunocompetent mice is generally intact until the 6^th^ day and its size is not influenced by inflammation during this time-window 6. Analysing a vast array of neoplasms with different histological subtypes, Levi *et al.* depicted preserved tumor composition with insignificant lymphocyte migration encountered on the 4^th^ day of SRCA 7.

Given these inherently unfavorable immunological effects possibly occurring in case of athymic nude mice xenografted with human tumors, focus was placed on the application of other alternatives 4. Although, neonatal thymectomy together with the administration of antithymocyte serum or thymectomy after whole body irradiation and bone marrow reconstruction may grant ideal circumstances for the development of human tumor xenograft in mice, taking their demanding procedure into consideration, such immunosuppressed animals did not gain widespread use at preclinical level 4, 8. Together with the ever-increasing bunch of anticancer agents, studies with different implantation areas and examination times - as potential alternatives - were also underway 4. Therefore, in contrast to the original 11-day long methodology employing nude mice, later on the SRCA was developed for application with normal immunocompetent mice as well, and the length of the test was shortened to 6 days 4.

### Human-derived SRCA tumor models

In 1979 *Bogden and co-workers* reported a study on SRCA technique with human breast and colon carcinoma lines tested herewith in normal, immunocompetent mice 4. Attempting to prohibit the development of primary host immune response, - that is considered to occur on day 7 - in that series of experiments the assay period lasted for only 6 days instead of the previously described 11 4. Further, the drugs were distributed on days 1-5 or on days 1, 3 and 5 which is another notable distinction from the SRCA of 1978 4. Later in 1981 SRCA was intended to be established as a prospective method for the identification of tumor sensitivity to chemotherapeutic drugs 9. Several studies strengthened its efficacy. Bogden *et al.* recounted the feasibility of the 6-day SRCA with the application of immunocompetent animals 4. Promising results were published with primary surgical explants as well, presenting progression more commonly applying SRCA with the shortened time frame using immunocompetent animals compared to the 11-day long method with nude athymic mice 4. Kangas *et al*. concluded that 6-day SRCA could be utilized for the chemotherapeutic response evaluation of human breast tumors 10. Further, this technique was assumed to be applicable to ovarian cancers as well 11.

In 1983, *Edelstein and colleagues* claimed that the development of the implanted but not treated controls in the 6-day SRCA was affected by the homogeneity of the donor tumor 12. This finding re-ignited the interest in the in-depth investigation of tumor structure. Early histological examinations pointed out the variability of the examined tumor fragments in the amount of fibrosis and the inward growth of host cells 13. The extent of host cell growth into the tumors highlights the insignificant amount of histologically proven malignant cells inside the implantable fragments 5.

Since physiological host immune reaction can result in the measurement of the chemotherapeutic agent induced immunosuppression instead of the desired antitumor effect of the molecule, SRCA was further developed by the administration of anti-inflammatory molecules, cortisone pre-treatment, or whole-body irradiation prior to the implantation itself 5, 14. Data are available about the necessity of the application of either pre-implantation treatment or other modifications to inhibit the occurrence of host cell ingrowth into the tumors 3. *Edelstein and co-workers* laid down that a vast array of immunosuppressive agents may decrease the extent of host cell ingrowth, however, there is still much to be done to fully overcome this difficulty 14. Additionally, due to the decrease in the preoperative irradiation enhanced immunosuppression by day 6, Edelstein *et al.* recommended the introduction of a treatment regime to gain reliable measurements on the anti-proliferative effects of the tested drugs 5. The effectivity of further modifications of the initial technique was tested in other studies. *Bennett and co-workers* also strengthened the demand for the adjustment of pre-medicalization 15. In addition, they confirmed host cell invasion without pre-treatment in normal, immunocompetent mice 15. In contrast to the original SRCA proposed by Bogden, the assay time frame applied by Levi *et al.* was only 4 days 16. A further change was the utilization of cell lines instead of primary tumors 17.

All in all, the subsequent early results confirm the suitability of the SRCA method in the rapid screening of the therapeutic evaluation of anti-tumor drugs. In a study conducted by Bogden *et al.* - based on both laboratory and clinical data - the 6-day SRCA was reported to be a valuable predictive test system for the development of medications and different treatment options at both clinical and preclinical levels 18. It was also added that tumor heterogeneity does not influence the performance of SRCA 18. However, preliminary results indicate that SRCA only suits for quite homogenous and rapidly developing tumor types 3. To ensure the maximum efficacy of the medication within the assay time, Edelstein *et al.* considered those particular neoplasms to be amenable for SRCA drug analysis which presented augmented growth pattern 5. Pulmonary small cell tumors were supposed to be potential candidates for this technique 3. In an article published by Edelstein *et al.* in 1985, SRCA was considered to have restricted efficacy for screening purposes and small usefulness for prospective assessment 5. According to other results, the efficacy of the 6-day SRCA and the functionality of immunocompetent mice outline its feasibility to test the anticancer effects of drugs against a broader spectrum of human malignancies 4. Individual tumor response may also be defined applying SRCA with primary surgical explants 4. For example, better response and enhanced tumor shrinkage were experienced in tumors with rapid progression compared to the ones with slow growth potential in a study investigating 75 ovarian and 71 pulmonary tumors 2. These results confirmed the proficiency of SRCA regarding the identification of the variability of drug sensitivity of tumors with different growing profile.

### Fading of the original SRCA method

Despite the above detailed numerous advantages of the SRCA method, the technique was quickly faded in the 1990s. To our best knowledge, the exact reason why SRCA method faded in the 1990s is not known. However, there are some reasons which could be the reasons behind. We suppose that anti-implantation effects of the tested drugs, xenograft-associated host immune response, and the variability of the implanted tumor fragments were initial difficulties that possibly decelerated the spread of SRCA. Moreover, we further speculate that the heterogeneity of the transplanted tumors and the different growing potential made only some types of malignancies to be amenable for SRCA screening.

From technical point of view there are some drawbacks of the SRCA technique that need to be considered such as: the challenging characteristics of the operation, the special circumstances required for the execution of the whole process and subsequent financial burden. These could also provide a reasonable explanation for the rapid fading of this method.

## Syngeneic SRCA tumor models

The SRCA method proposed by Bogden *et al.* primarily aimed at evaluating the efficacy of anti-cancer drugs (seen in **Table 1**). Since the development of animal tumors is identical to that of human malignancies, *in vivo* animal experimental models represent promising translational systems in the assessment of tumor growth and metastasis progression.

Therefore, the SRCA technique was utilized for the adequate preclinical investigation of the kinetics of tumor progression and metastatic spread by *Uzvolgyi and co-workers*, where the transplantation of a predefined number of Myelomonocytic Leukemia (My), Mesoblastic Nephroma (Ne) and Hepatocellular Carcinoma (He) cells - derived from chemically induced tumors - was transplanted under the left renal capsule of rats, thereby a new syngeneic animal tumor model was established 19.

Since SRCA-based previous histological research data revealed alterations in parathymic lymph nodes with the appearance of neoplastic cells inside, this renal capsule/parathymic lymph node complex was supposed to be an ideal tool for the in-depth understanding of the spread of atypical cells, thus the route of metastatisation of primary tumors and the formation of secondary malignancies 20. The research team of *Kertai and Trencsényi* - as will be detailed in the publications presented later - had a pioneering role in the development and integration of the renal capsule/parathymic lymph node complex in the field of nuclear medicine at preclinical level. Using positron emission tomography (PET) tracers, mainly deoxy-2-[^18^F]fluoro-D-glucose ([^18^F]F-FDG) and [^11^C]C-methionine they have managed to identify both the primary tumors and their metastases in different experimental animals transplanted with various tumor cell types. Their initial experiences laid the basis of SRCA-related potential future drug development and radiopharmaceutical testing (**Table 1**).

### SRCA - the operation

**Figure 1:**
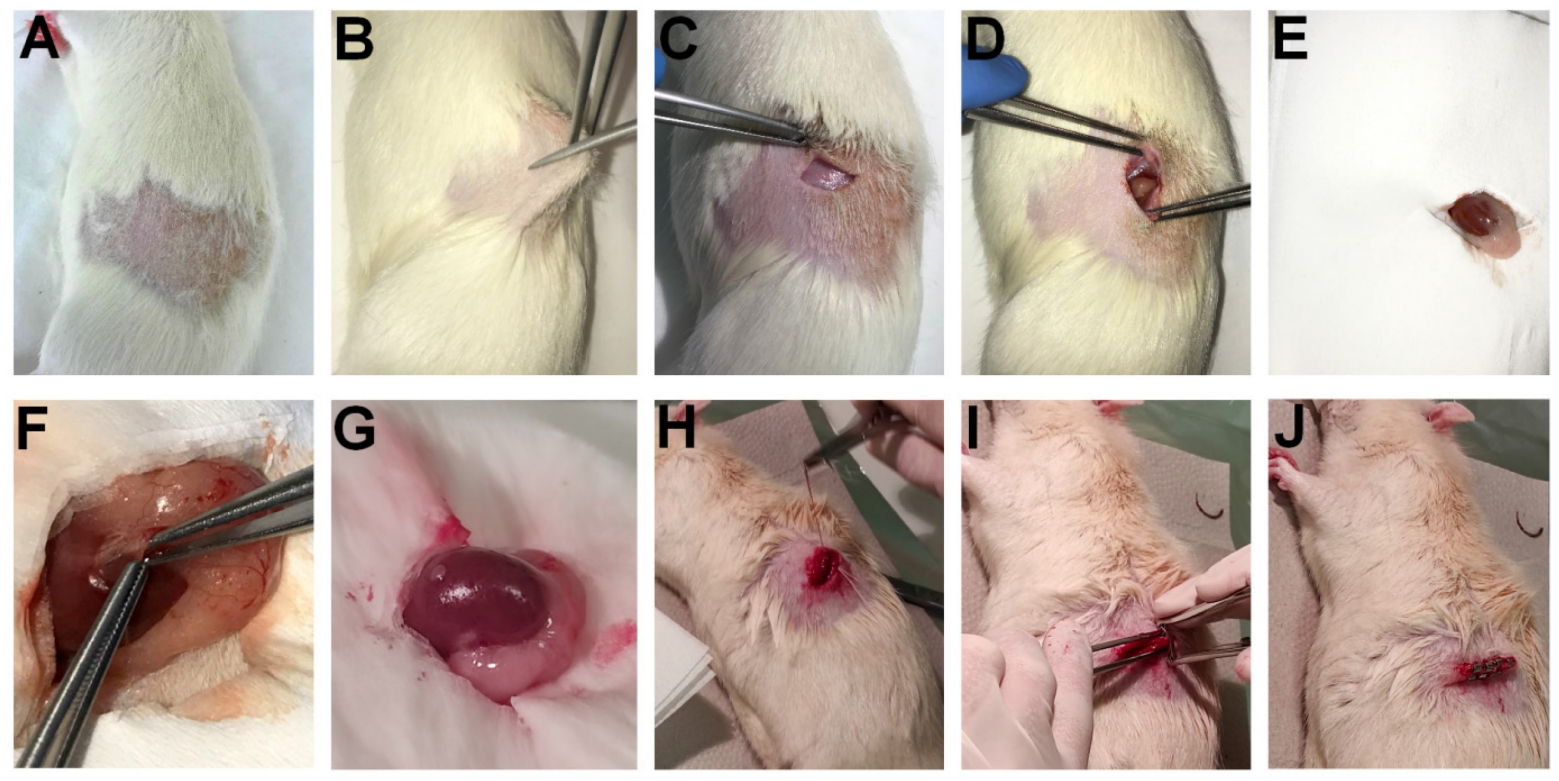
**Step-by-step demonstration of the process of SRCA: 1A:** Shaving the fur off the lumbar region of the experimental animals one fingerbreadth under the ribs on the left. Desinfection of the area of concern. **1B-1D:** Intersection of the skin and the muscle layer to open the retroperitoneum and to reach the left kidney. **1E:** Continuous hydration of the uncovered left kidney with physiological saline solution (the perirenal fat is also clearly visible).** 1F:** Exposure of the left kidney and the placement of tumor cell-containing disc in 10 μL physiological saline solution (0.9% NaCl solution) under the left renal capsule of the experimental rats through a small incision performed applying Iris scissors. **1G:** The exposed left kidney with the tumor cells already implanted under the renal capsule. **1H-1I:** Suture of the muscle layer. **1J:** Closure of the skin layer with the application of surgical staples.

In 2009, *Kertai and Trencsényi* profoundly described the SRCA procedure in rats 21. Tumor fragments or cancer cells placed on Gelaspon^R^ discs were implanted under the capsule of the left kidney of the experimental animals. Gelatin sponge discs with a diameter of 4 mm and a thickness of 1 mm were prepared using Gelaspon^R^ gauze (Germed, Rudolstadt, Germany) and were sterilized to carry out cell transplantation. This was followed by the deposition of the neoplastic cells in 10 μL saline (0.9% NaCl solution) on the Gelaspon^R^ discs 21. Rats were anaesthetized before the surgery by intraperitoneal *(i.p)* administration of 3 mg/100g pentobarbital (Nembutal, Ceva-Phylaxia RT. Budapest, Hungary). They shaved the fur off the lumbar region of the animals one fingerbreadth under the ribs on the left, and the area was desinfected. The skin and the muscle layer were intersected to open the retroperitoneum and to reach the left kidney. The uncovered left kidney was continuously moisturized with physiological saline solution. Then, the kidney was exposed, and a small incision was performed on the capsule renalis using Iris scissors, through which tumor cell-containing disc in 10 μL physiological saline solution (0.9% NaCl solution) was placed under the renal capsule of the experimental rats. Finally, the muscle layer was sutured, and the skin layer was closed with surgical staples. The steps of the SRCA procedure are presented in **Figure 1**.

### Overview of nuclear medical research studies applying the SRCA-induced Renal capsule/Parathymic lymph node complex

**Figure 2:**
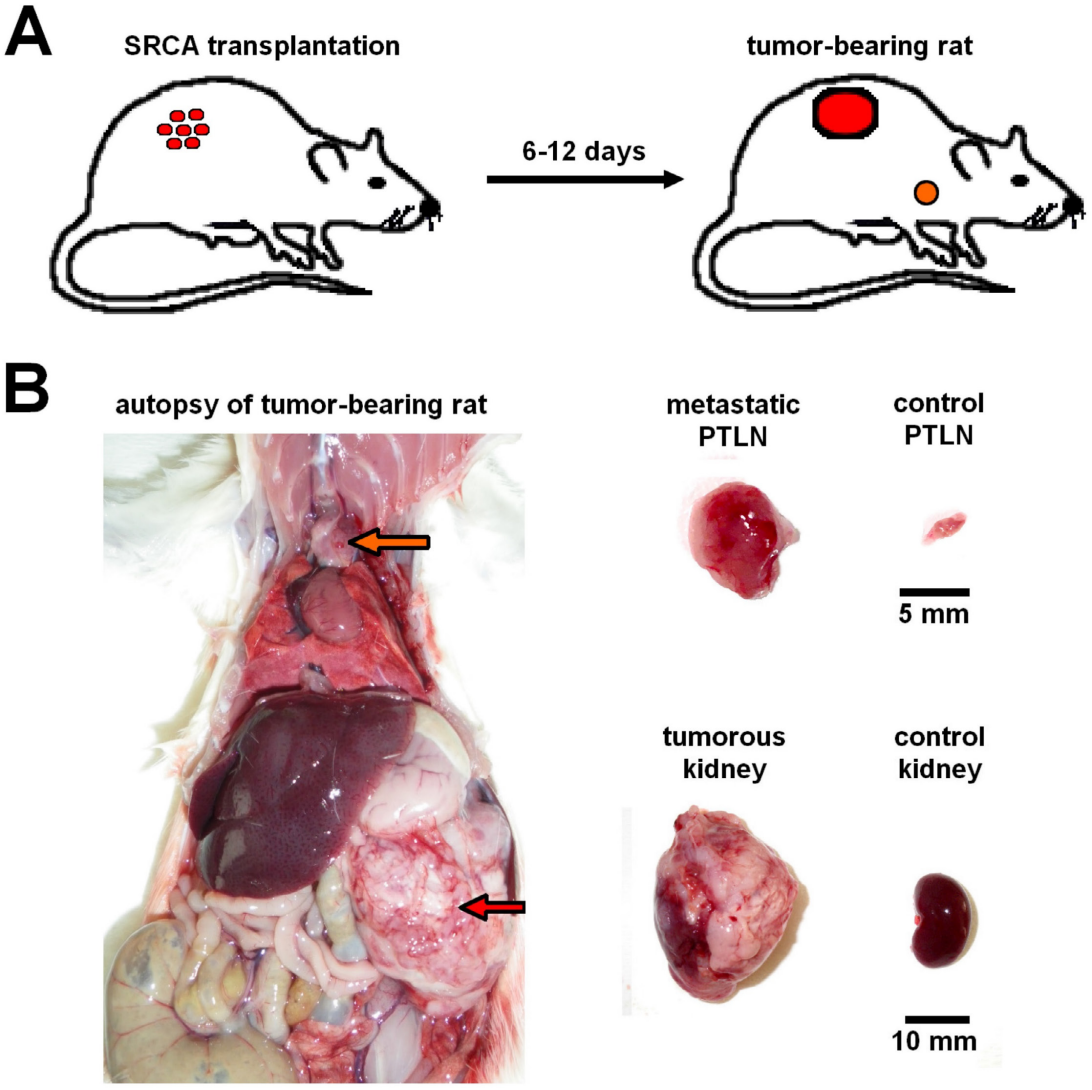
** Presentation of renal capsule/parathymic lymph node complex: 2A:** demonstrates the process of the development of primary tumors 6-to-12 days after the transplantation of tumor cells (He/De, Ne/De, My2/De, My1/De) under the left renal capsule with the application of SRCA method as well as the formation of metastasis in the PTLN of the tumor-bearing rat. **2B:** The right side of the figure presents the autopsy of a tumor-bearing rat. The upper arrow (orange) shows the metastatic PTLN, whereas the lower arrow (red) demonstrates the subrenally localised primary tumor. On the left side a metastatic PTLN, and a tumorous left kidney could be visualised separately compared to healthy control organs.

With the application of different neoplastic cell lines several experiments were conducted to strengthen the suitability of the SRCA method in the assessment of metastases formation. In a study executed by *Trencsényi and co-workers* in 2009, preclinical small animal PET imaging (miniPET), whole-body autoradiography and *ex vivo* organ distribution investigations were applied to verify that rat Hepatocarcinoma (He/De) and Mesoblastic Nephroma (Ne/De) cells metastasize to the parathymic lymph nodes (seen in **Figure 2**). Using SRCA method 10^6^ He/De or Ne/De cells were implanted in the left subrenal region. Based on the results, both He/De and Ne/De tumors - developing under the left renal capsule - were assumed to display a remarkable metastatic load in the parathymic lymph nodes. This research also proves the feasibility of this renal capsule/parathymic lymph node complex in the separate *in vivo* evaluation of tumor metastatisation and the assessment of secondary tumors as well.

The previous findings were further corroborated by the same research group 21. Employing SRCA in rats, Epithelial Hepatocarcinoma (He/De), Mesenchymal Mesoblastic Nephroma (Ne/De) cells, and tumor-bearing lymph nodes were placed under the left renal capsule of F344 rats to dissect metastasis formation by [^18^F]F-FDG whole-body autoradiography and phosphor image analysis within the framework of establishing an *in vivo* preclinical model 19. Organ distribution studies were also performed after the gamma counter-based (Canberra Packard) registration of the radioactivities (expressed as differential absorption ratio/DAR) of the tissue specimens taken from the tumor, the kidney, the thymus, the musculus rectus abdominis and the parathymic lymph nodes. Mesenteric lymph node enlargement, angiogenetic processes, and the development of metastasis in the parathymic lymph nodes were experienced. These results draw the attention to the invasion of the parathymic lymph nodes as well as aggravated pyruvate kinase activity 20, 22. In line with their prior results, [^18^F]F-FDG uptake of the primary tumors and the parathymic lymph nodes outlines that He/De and Ne/De tumors spread to the parathymic lymph nodes. This metastatic potential was further strengthened by the observation that the extracted and subrenally placed parathymic lymph nodes were comparable to the primary tumors six days after the growth of He/De or Ne/De neoplasms. Further, the lymphatic relationship between these lymph nodes and the lymphatic vessels of the left kidney capsule was also perceived by the presence of India Ink in the lymph nodes one day after its subrenal implantation. Prior literature data on research with staining and contrast X-ray examinations set that the peritoneal cavity possesses a lymphatic vasculature - consisting of three subsets - that crosses the diaphragm and is located on its thoracic part. The retrosternal one, that directs lymph to the upper mediastinal lymph nodes is in the proximity of the arteria mammaria interna 23, 24, 25. In 1971, the lymphatic drainage of the rats was profoundly described by *Tilney* 26. According to his publication the lymph of the parathymic nodes originates from the peritoneal cavity, the pericardium, the liver, and the thymus and is directed towards the mediastinal lymph trunk. Thus, we suppose that followed by the invasion of the lymphatic vessels of the diaphragm, the subrenally located tumor cells infiltrate the parasternal lymphatic vessels before entering the parathymic lymph nodes. In conclusion, this study also emphasizes how promising this renal capsule/parathymic lymph node complex is regarding the *in vivo* analysis of metastasis progression as well as the formation of secondary neoplasms 21. In addition, the established model might grant an opportunity to investigate tumor cell migration from the subrenal area to the parathymic lymph nodes, that could have human implications in terms of therapeutic management.

Later, another study dealing with subrenally localized mesenchymal renal tumor cells (Ne/De) obtained from metastatic parathymic lymph nodes also clarified the relationship between the lymphatic vasculature of the renal capsule and the parathymic lymph nodes 27. In the same study the [^18^F]F-FDG radiopharmaceutical uptake pattern of the organs also corroborated that the parathymic lymph nodes are the first sites in tumor metastatisation (as demonstrated in **Figure 2**). Further, histological examinations provided the following profound evaluation of the dynamics of tumor progression. At early stages, normal and tumorous kidney parenchyma could be definitely differentiated particularly in the periphery of the tumor mass. During the tumor development infiltration rather than invasion became dominant leading to the rupture of the tissue of the kidney. Given the absence of angiogenesis, the tumor niche was characterized by increasing amounts of lipid substances and an enhancement of blood supply to the lymphatic drainage system. Thus, the resultant elevated interstitial pressure triggered the ingrowth of neoplastic cells into the parathymic lymph nodes.

Based on data from 2012, renal capsule/parathymic lymph node *in vivo* metastatic rat model was again used to study tumor invasion in Fisher 344 rats transplanted with N-nitrosodimethylamine-triggered He/De and Ne/De cells under the left renal capsule 28. Besides the appearance of neoplastic cells in the parathymic lymph nodes, *i.p.* administered tumor cells were identified in other thoracic lymph nodes including the mediastinal internal mammary ones. This could be explained by the fact that malignant cells may enter either anterior mammary or parathymic lymph nodes through the transdiaphragmatic drainage.

In order to compare the SRCA technique with other conventional methods of tumor initiation, studies that simultaneously applied different procedures were also underway.

**Figure 3:**
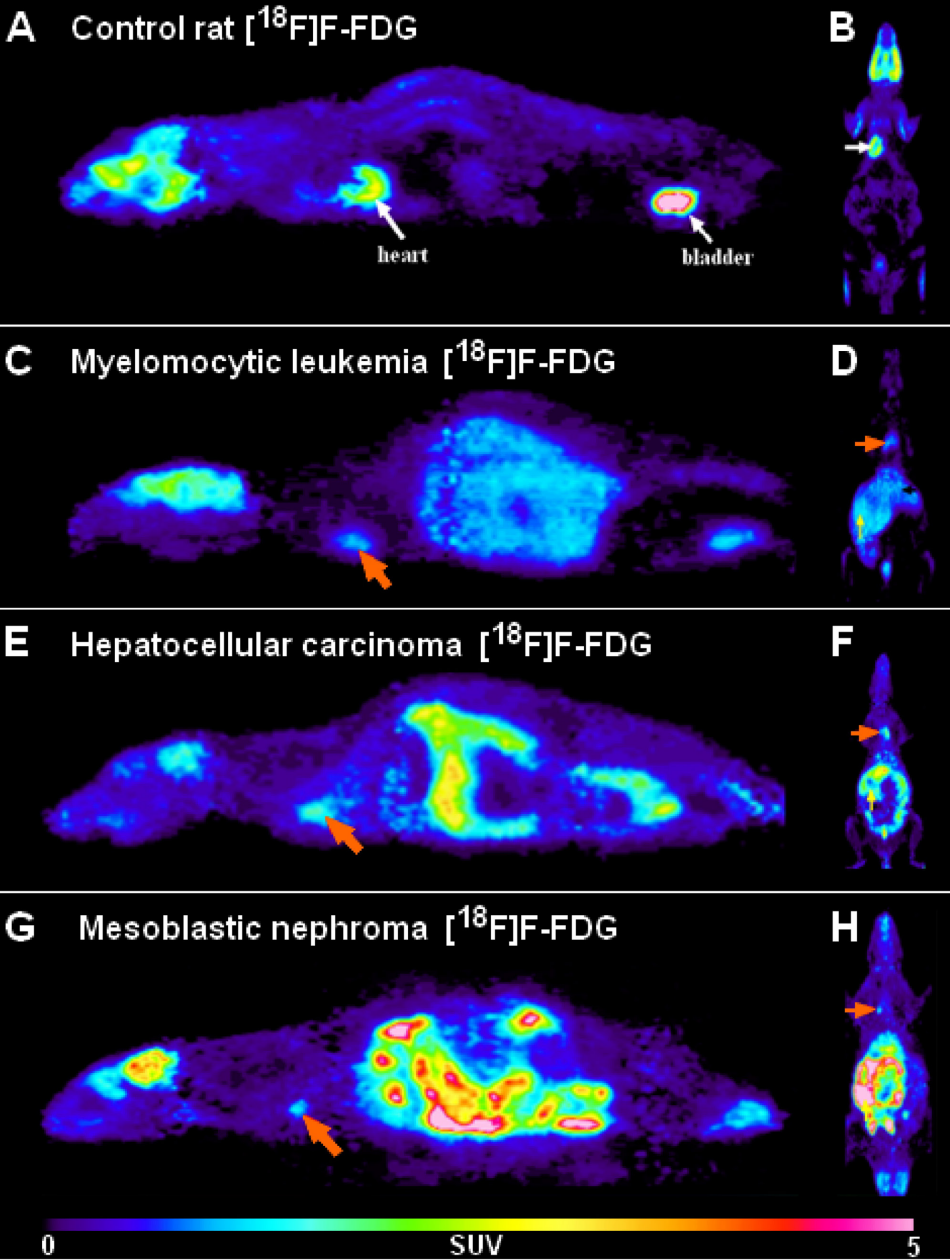
Representative [^18^F]F-FDG PET images of healthy control rats (***A, B***) and the tumor-bearing experimental animals transplanted with My2/De (***C, D***), He/De (***E, F***) and Me/De (***G, H***) tumor cells under the left renal capsule applying the SRCA method. **2A** and** 2B** present the high physiological tracer accumulation of the brain, heart (*white arrows*) and the urinary bladder (*white arrows*) in sagittal (left) and coronal (right) orientation, respectively. In **2C, 2E** and** 2G** the sagittal [^18^F]F-FDG PET slices of the tumorous rats show intense and inhomogeneous retroperitoneal radiopharmaceutical uptake of the primary tumors, whereas the physiologically accumulating organs are identified with a more discreet activity compared to the control animals. The *orange arrows* point to the parathymic lymph node metastasis. Similarly, in 2**D, 2F,** and** 2H** the elevated uptake of the parathymic lymph node metastasis (*orange arrows*) and the increased radiopharmaceutical accumulation of the subrenally localised malignancies are demonstrated in coronal orientation.

With this attempt Trencsényi *et al.* in 2014 carried out the subcutaneous (*s.c.*)*,* intravenous (*i.v.*) injections, and SRCA-based syngeneic implantation of He/De cell lines utilizing Fischer 344 rats to observe which way of tumor induction bears the best performance in terms of the investigation of tumor growth and metastatic progression 29. Followed by the administration of urethane overdose, 6, - 12, - and 18 days after euthanization autopsy was completed. Then, [^18^F]F-FDG PET was employed for the analysis of tumor development and the pathway of metastatisation. A representative PET scan is shown in **Figure 3**. Tumor tracer uptake was provided in standardised uptake value (SUV) units and based on the ratio of tumor SUV mean and background muscle SUV mean, tumor to mediastinal (T/M) ratios were also presented as qualitative data. Given the enhanced expression of various proteins on tumor cell membranes, for the verification of *in vivo* PET findings, glucose transporter 1 (GLUT-1), GLUT-3, and transforming growth factor beta 1 (TGF- ß1) expressions were measured in the tumor-bearing and the healthy kidneys 14 days post-transplantation 30, 31. The results of PET image interpretation and postmortem experimental data led to the recognition that *s.c.* injected tumor cells did not generate metastasis, therefore this form of tumor induction is not applicable for metastasis screening. However, literature data strengthen the feasibility of other *s.c.* tumor models regarding metastasis formation in distant localizations 32. After the *i.v.* injection of tumor cells, pulmonary and hepatic tumorous masses could be registered. Although *i.v.* tumor induction led to metastasis projection in distant visceral organs, due to several precariousness, this method did not gain widespread clinical use. First of all, the unreliability of the method could be confirmed by the result according to which only three out of five rats demonstrated metastasis formation. Further, compared to the *s.c.* administration, slower speed of tumor development with smaller tumor sizes could be detected. In addition, to avoid pulmonary microembolization, *i.v.* injected tumor particles must be within the colloidal size range. Finally, injury to the caudal vein results in *in situ* tumor formation that is another unfavorable feature of the *i.v.* method. In accordance with prior results of SRCA-associated research, He/De tumor cells triggered metastasis formation first in the parathymic lymph nodes. In the present study, postmortem surgical explorations also evidenced the parathymic lymph nodes to be the sentinel lymph nodes of the He/De tumors. Besides the extreme enlargement of these tumor-bearing lymph nodes (23.5±2.5 mg to 1.5-2.0 g), disorganization and red blood cell invasion were depicted in their histological samples. *Trencsényi and co-workers* concluded that with the application of SRCA, tumor growth could be achieved at a high speed and the development of metastasis occurred in all animals. They also summarized the route of tumor cells from the retroperitoneum to the thorax. Briefly, in the first place late-onset angiogenesis causes necrotization inside the tumor milieu. Due to changes in the physiology of the vasculature, blood cells and neoplastic cells accumulate in the interstitial fluid leading to an increase in its pressure 28. Consequently, neoplastic cells are directed towards the surrounding tissues through the increased pressure-linked external damage of the tumors 33. Then, the released cells pass from the primary renal tumor to the retroperitoneum, and from the liver to the peritoneal cavity, and then to the diaphragm, the thoracic lymphatic vasculature and finally to the parathymic lymph nodes 21, 34, 35.

Besides He/De and Ne/De cell lines, Myeloid Myelomonocytic Leukemia 2 (My2/De) and Myeloid Leukemia 1 (My1/De) cells were also utilized for the SRCA-based investigation of tumor progression and metastases development.

Trencsényi *et al.* in 2014 used inbred Long Evans rats to thoroughly explore the *in vivo* metastatic spread of My2/De leukemia cells with the application of [^18^F]F-FDG or [^11^C]C-methionine 36. The metastatic invasion was followed by the administration of the radiopharmaceuticals via the tail vein, and the subrenal or *i.p.* employment of India Ink. His research team also intended to make a distinction between the hematogenous, and the lymphatic spread of My2/De cells after implantation. With the SRCA method, 10^6^ My2/De cells in 10 μL saline on Gelaspon^R^ disc were placed in the left subrenal region of the experimental rats. Within the framework of *in vitro* radiotracer accumulation studies, 4 weeks post-implantation, followed by preincubation in phosphate buffered saline (PBS) supplemented with 5 mM D-glucose at 37 °C for 10 min, bone marrow cells (10^6^) of both healthy control and tumor-bearing rats were treated with 0.37 MBq/mL [^18^F]F-FDG and 1.85 MBq/mL [^11^C]C-methionine. After repeatedly washing the cells 3 times with ice cold PBS, a gamma counter (Canberra, Packard) was applied to adequately determine the 1-minute radiopharmaceutical uptake of the cells in counts per min (cpm) units/10^6^ cells. *In vivo* metastatic propagation was evaluated one month after SRCA by performing 20-minute static one-frame PET scans (animal MiniPET-II scanner) followed by the administration of both tracers through the tail vein of the healthy and the leukemic rats. This enabled the quantitative determination of tracer uptake provided in SUV units. **Figure 3** presents representative PET scans of My2/De tumor-bearing rat. Tissue distribution was defined in DAR units - (accumulated radioactivity/g tissue)/(accumulated radioactivity/g tissue) - by the measurement of the weight and the radioactivity of the tissues. In order to fully confirm the metastatic route of the My2/De cells, 0.5 mL India Ink (Gunther Wagner, Pelikan Werke, Hannover, Germany) was either *i.v.* or *i.p.* diffused. In conformity with prior research findings with Ne/De and He/De tumor cells, *Trencsényi and co-workers* observed that the subrenally placed My2/De tumors also metastasize to the parathymic lymph nodes 28. While My2/De cells growing under the capsule of the left kidney spread to visceral abdominal organs and to the parathymic lymph nodes, these nodes remained intact during the hematogenous propagation of the leukemic cells. Similarly, in case of the *i.p.* injection of the malignant cells, the parathymic lymph nodes proved to be the primer sites of metastatisation. In line with these results, the same phenomena were experienced with the application of India Ink that further strengthened the route of metastatisation.

In a work from 2015, *Trencsényi and co-workers* used another cell type, namely My1/De cells derived from DMBA-induced Myelomonocytic Leukemia to transplant under the left renal capsule of male Long-Evans LBF1 rats applying the same SRCA technique that was previously described 37. Besides liver, spleen and parathymic lymph node enlargement, cytology revealed approximately 10 times higher amounts of white blood cells comprising 30-50% of blast cells in the blood smear of the tumor-bearing rats in comparison with normal controls two-weeks post-implantation. My1/De cell invasion of the liver and spleen was supported by histological data. For the further assessment of the tumorigenic potential of the leukemic cell culture, besides cytology, the kinetics of [^18^F]F-FDG accumulation of My1/De cells, control bone marrow cells and My1/De bone marrow cells were compared. My1/De cells expressed noticeably more elevated tracer uptake than those of the cells of the bone marrow. The 100% blast cell content of the leukemic cell culture and the heterogenous cell population of the bone marrow with various glucose needs may provide a possible interpretation for this finding 37.

## Conclusive remarks

We presuppose that the aforementioned renal capsule/parathymic lymph node complex resembles the Ranke complex - including a peripheral pulmonary alteration and mediastinal lymph nodes developed after TBC infection/inflammation. We anticipate that SRCA will gain foreseeable widespread applicability at both nuclear medical preclinical and clinical levels, particularly in oncological fields. In-depth assessment and expanded knowledge of angiogenesis, tumor propagation and metastasis development provided by SRCA-based investigational scenario would grant the opportunity to seek novel anti-cancer therapeutic targets and reliable isotope diagnostic biomarkers. This could further lead to the proposal of new drug-to-be anti-tumor molecules and radiopharmaceutical development. In addition, the follow-up of a variability of consecutive neoplastic generations by the subrenal transplantation of parathymic lymph nodes of the tumor developments is also possible applying nuclear medical imaging methods.

Further, the whole process of the metastatic cascade from primary tumor formation to the appearance of metastasis itself can be followed by non-invasive nuclear medical imaging techniques (**Table 2**). Since deeper understanding of the metastatic cascade may lead to changes in currently used human cancer treatment regimes more individualised patient care could be achieved with the integration of SRCA-derived research data into clinical therapeutic decision making. These novel clinical implications may revolutionize the already existing oncological treatment guidelines as well as the diagnostic armamentarium of neoplastic diseases. All in all, SRCA and related research evidence eventually may lead to the establishment of personalised oncological treatment. As for preclinical aspects, animal model systems could be valuable regarding trials of drug candidates and radiopharmaceutical testing as well as development.

In summary, we anticipate the promising re-emergence of SRCA at preclinical level as it would be of greatest significance in the development and testing of targeted radiopharmaceuticals leading to the better assessment of molecular mechanisms of various neoplastic diseases.

## Figures and Tables

**Table 1:** Comparison of the most important characteristics of the original and re-emerged SRCA

	Original SRCA	Re-emerged SRCA
Aim	Drug testing	Investigation of tumor formation, and development in nuclear medicine
Model type	Xenograft	Syngeneic
Experimental animals	Immunodeficient mouse	Immunocompetent mice, rats
Transplanted element	Human tumor sample	Tumor fragments, cancer cells
Volume of the element	1 mm^3^ (1x1x1)	Specific cell number
Localization	Left subrenal area	Left subrenal area
Neoplastic cell content	approx. 70%	100%
Duration of SRCA	11 days	min. 7 days
Metastasis formation	No	Yes
Drug administration	Yes	No

**Table 2:** Evaluation of primary and metastatic tumor progression and related processes by radiopharmaceuticals and nuclear medicine techniques in SRCA-induced preclinical tumor models

Tumor type	Target molecule	Investigated process	Radiopharmaceutical	Nuclear medicine imaging technique	Reference
Ne/De	APN/CD13, ανβ3 integrin, glycolysis	metastasis formation, tumor associated neo-angiogenesis	[^68^Ga]Ga-NOTA-c(NGR), [^68^Ga]Ga-NODAGA-[c(RGD)]_2_, [^18^F]F-FDG	*in vivo* PET	41
Ne/De	APN/CD13, ανβ3 integrin	primary tumor and metastasis formation, tumor associated neo-angiogenesis	[^68^Ga]Ga-NOTA-c(NGR), [^68^Ga]Ga-NODAGA-[c(RGD)]_2_	*in vivo* PET, *ex vivo* gamma-counting	38
My1/De	glycolysis, cell proliferation	primary tumor and metastasis formation, bone marrow analysis	[^18^F]F-FDG	*in vitro* gamma-counting	37
My2/De	glycolysis, cell proliferation	primary tumor and metastasis formation, bone marrow analysis	[^18^F]F-FDG [^11^C]C-methionine	*in vivo* PET, *ex vivo* and *in vitro* gamma-counting	36
He/De	glycolysis, cell proliferation	primary tumor and metastasis formation	[^18^F]F-FDG	*in vivo* PET	29
He/De	folate receptors, glycolysis	primary tumor formation and tumor targeting	[^18^F]F-FDG γ-PGA-FA/CH-AF-Gd nanoparticle	*in vivo* PET/MR	39
He/De	folate receptors, glycolysis	tumor targeting	[^99m^Tc]Tc-CH/γ-PGA-FA-NP nanoparticle	SPECT/CT	40
Ne/De, He/De	glycolysis, cell proliferation	primary tumor and metastasis formation and tumor targeting	[^18^F]F-FDG	*ex vivo* and *in vitro* gamma-counting, *ex vivo* autoradiography	33
Ne/De	glycolysis, cell proliferation	primary tumor and metastasis formation and tumor targeting	[^18^F]F-FDG	*ex vivo* gamma-counting	27
Ne/De, He/De	glycolysis, cell proliferation	primary tumor and metastasis formation and tumor targeting	[^18^F]F-FDG	*ex vivo* gamma-counting, *ex vivo* autoradiography	21

## Abbreviations

SRCA: subrenal capsule assay
My1/De: chemically induced Myelomonocytic Leukemia
My2/De: spontaneous Myeloid Leukemia
He/De: Epithelial Hepatocellular carcinoma
Ne/De: Mesenchymal Mesoblastic Nephroma
PET: positron emission tomography
[^18^F]F-FDG: 2-deoxy-2-[^18^F]fluoro-D-glucose
SUV: standardizes uptake values
T/M: tumor to mediastinal
PTLN: parathymic lymph node
